# *Paramecium*, a Model to Study Ciliary Beating and Ciliogenesis: Insights From Cutting-Edge Approaches

**DOI:** 10.3389/fcell.2022.847908

**Published:** 2022-03-14

**Authors:** K. Bouhouche, M. S. Valentine, P. Le Borgne, M. Lemullois, J. Yano, S. Lodh, A. Nabi, A. M. Tassin, J. L. Van Houten

**Affiliations:** ^1^ CEA, CNRS, Université Paris-Saclay, Institute for Integrative Biology of the Cell (I2BC), Gif-sur-Yvette, France; ^2^ SUNY Plattsburgh, Plattsburgh, NY, United States; ^3^ Department of Biology, University of Vermont, Burlington, VT, United States; ^4^ Biological Sciences, Marquette University, Milwaukee, WI, United States; ^5^ Luminex, Austin, TX, United States

**Keywords:** *paramecium*, basal body, transition zone, cilia, ciliary beating, ion channel, cryo-tomography

## Abstract

Cilia are ubiquitous and highly conserved extensions that endow the cell with motility and sensory functions. They were present in the first eukaryotes and conserved throughout evolution (Carvalho-Santos et al., 2011). *Paramecium* has around 4,000 motile cilia on its surface arranged in longitudinal rows, beating in waves to ensure movement and feeding. As with cilia in other model organisms, direction and speed of *Paramecium* ciliary beating is under bioelectric control of ciliary ion channels. In multiciliated cells of metazoans as well as paramecia, the cilia become physically entrained to beat in metachronal waves. This ciliated organism, *Paramecium*, is an attractive model for multidisciplinary approaches to dissect the location, structure and function of ciliary ion channels and other proteins involved in ciliary beating. Swimming behavior also can be a read-out of the role of cilia in sensory signal transduction. A cilium emanates from a BB, structurally equivalent to the centriole anchored at the cell surface, and elongates an axoneme composed of microtubule doublets enclosed in a ciliary membrane contiguous with the plasma membrane. The connection between the BB and the axoneme constitutes the transition zone, which serves as a diffusion barrier between the intracellular space and the cilium, defining the ciliary compartment. Human pathologies affecting cilia structure or function, are called ciliopathies, which are caused by gene mutations. For that reason, the molecular mechanisms and structural aspects of cilia assembly and function are actively studied using a variety of model systems, ranging from unicellular organisms to metazoa. In this review, we will highlight the use of *Paramecium* as a model to decipher ciliary beating mechanisms as well as high resolution insights into BB structure and anchoring. We will show that study of cilia in *Paramecium* promotes our understanding of cilia formation and function. In addition, we demonstrate that *Paramecium* could be a useful tool to validate candidate genes for ciliopathies.

## Introduction

Most of us have experienced the wonderful sight of ciliates swimming around in drops of pond water as we get to use a microscope for the first time. The cells swim forward, turn around, and zip out of the field of view. The chances are excellent that the prominent ciliate you saw was a *Paramecium*. Their swimming behavior powered by their thousands of cilia is what draws many of us to use *Paramecium* species to study how cilia work and how they are formed. It is no wonder with about 4,000 cilia per cell, the *Paramecium* presents the opportunity to study the form, function and positioning of cilia on the cell surface.

To swim forward, cilia on *Paramecium* beat with their power stroke toward the posterior of the cell and a lazy return stroke toward the anterior. When the power stroke reverses in response to some environmental stimuli, the power stroke is toward the anterior and return stroke toward the posterior pole, causing the cell to swim backward briefly (see (Satir et al., 2014) for a review).

**FIGURE 1:**
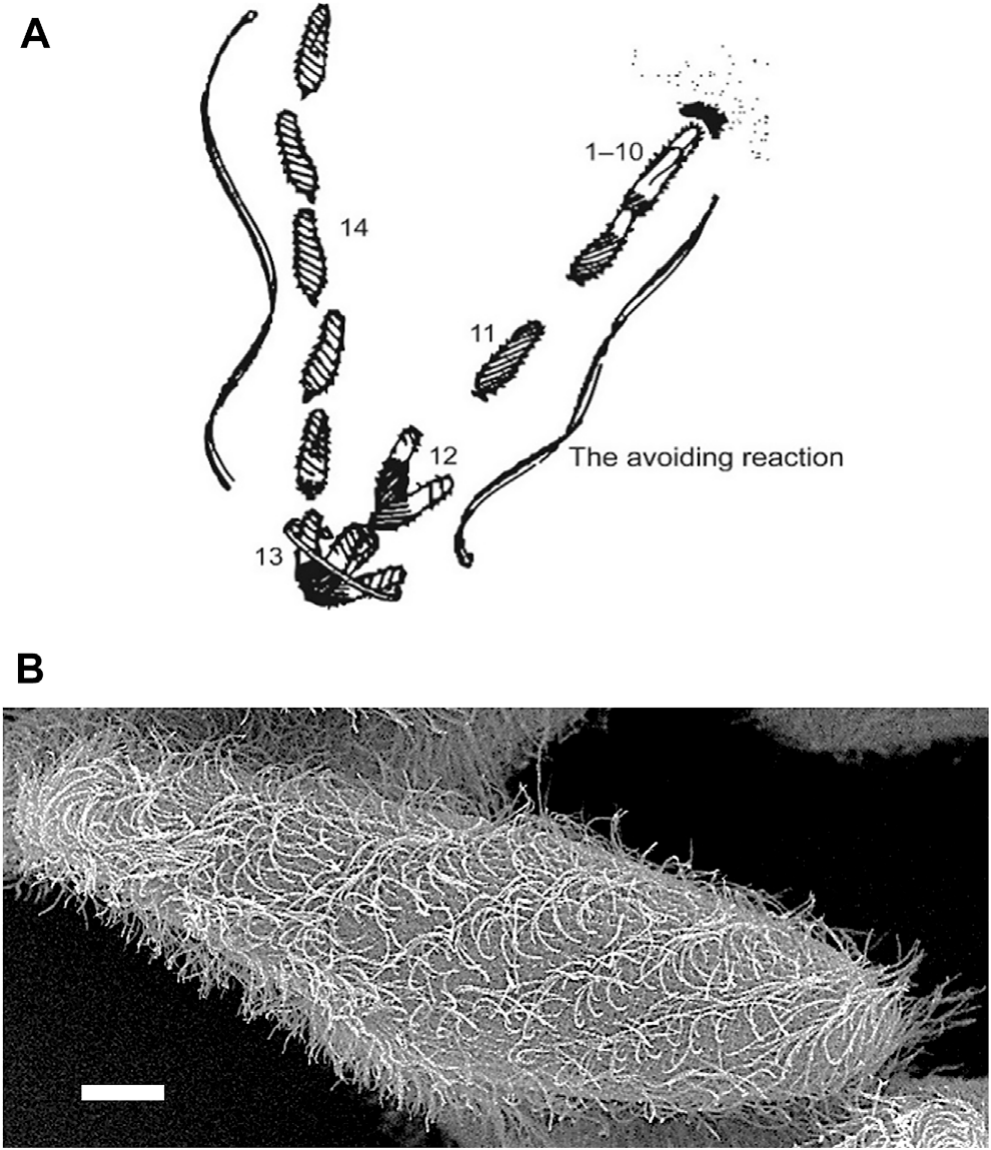
**(A)** An image based on the sketch of the stages of the avoiding reaction drawn by Jennings (Jennings, 1906). Anterior mechanical stimulation by a cell swimming into an object leads to depolarization, opening of the Ca_V_ channels of the cilia, movement of the cell backward for a short time, twirling in place, and forward movement in a new direction. From Eckert (Eckert, 1972). **(B)** Scanning electron micrograph image of *Paramecium* showing metachronal waves of cilia (Courtesy of M. S. Valentine.).

As the cell recovers its forward motion, it usually changes its swimming path thus making a turn (Figure 1A). Special motor dyneins move the cilia in graceful arcs of the power stroke and lazy return stroke. Figure 1B shows a cell with metachronal waves of cilia as it swims forward (Brooks & Wallingford, 2014). These waves are physically constrained and keep the cilia from tangling or interfering with one another. Note that within these waves, the cilia can be seen in stages of power and return strokes. The frequency of the beat and the change in power stroke are under bioelectric control involving ion channels mostly located in the ciliary membrane.

**FIGURE 2:**
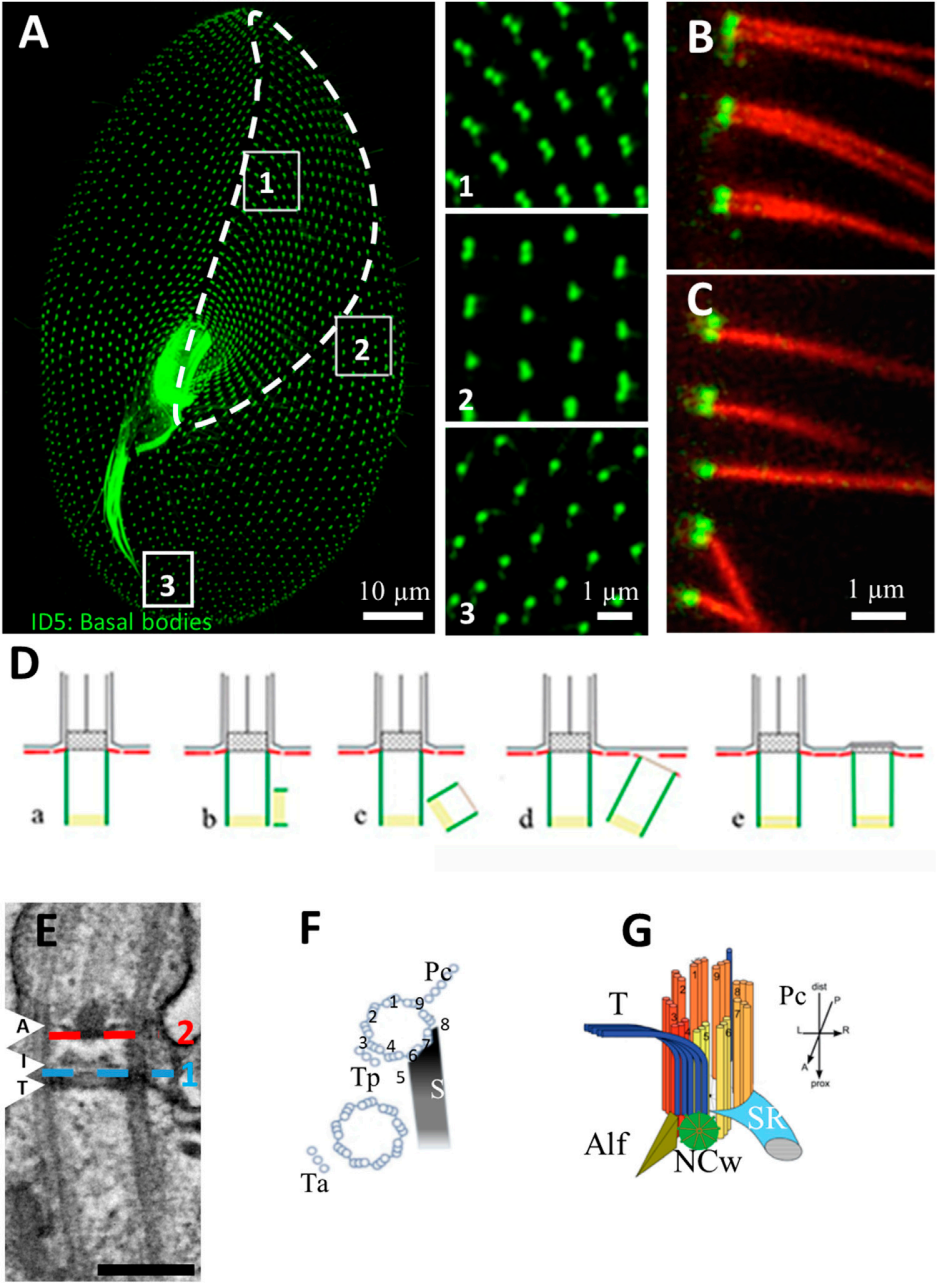
**(A)** Basal body organization in *Paramecium tetraurelia*. Basal bodies (BB) are decorated by monoclonal anti-glutamylated tubulin ID5 antibodies (green). Note that in the anterior part of the cell (area 1 corresponding to the invariant field, traced in white), basal bodies are organized in doublets. In the posterior part of the cell, singlet basal bodies are observed (area 3). In the rest of the cell, doublets or singlets of basal bodies are observed (mixed field, area 2). Scale bar: 10 µm. **(B,C)** Immunofluorescence showing basal bodies decorated by ID5 antibodies (green) and cilia stained by anti-polyglutamylated tubulin (red). Transverse section showing that in the invariant field (area 1), each BB, which are organized in doublets, grow a cilium. Scale bar: 1 µm. **(D)** Schematic representation of the different steps **(A–E)** of BB duplication. **(E)** Longitudinal section of a ciliated basal body observed by transmission electron microscopy Scale bar: 200 nm. The arrows indicated the position of the three layers of the TZ. Terminal plate (T), intermediate plate (I) and axosomal plate **(A)**. The dotted lines indicate the position of the transition fibers (1) and Y-links (2). **(F)** Schema of the BB appendages in a 2BB unit. The striated rootlet (SR) courses toward the anterior of the cell while the post-ciliary rootlet (Pc) is located near the 8th and 9th microtubule triplets. Also shown are the transverse rootlets on the anterior BB (Ta) and the posterior BB (Tp). **(G)** 3D representation showing the topographical relationships of BB and its anterior appendages: the striated ciliary rootlet (SR), the transverse microtubules (T) and the anterior left filament (ALF). The new BB (green disc). Reproduced/adapted with permission Aubusson et al, 2012. Journal of cell science, 125, 4395-4404 from Jerka-Diadosz et al., 2013.

Cilia emanate from basal bodies (BB), i.e., modified centrioles, anchored into the epiplasm, which is a submembranous skeleton segmented into cortical units. The spatial organization of a BB is defined as parallel longitudinal rows with a highly precise overall pattern (see Figures 2A–C). Unlike metazoa, there is no centrosome stage in paramecia, since their BBs always remain anchored at the cell surface. New BBs develop from the docked ones. Once duplicated, they just have to tilt-up to anchor directly at the cell surface, with axonemal elongation occurring afterwards (Figure 2D). During the docking process, as for mammalian cilia, the transition zone (TZ) assembles and displays its characteristic features: transition fibers and Y-links. The TZ comprises the structural junction between the BB and the nascent cilium (Gonçalves, J. & Pelletier, 2017) (Figure 2E). This TZ plays a crucial role in the biogenesis and function of cilia by forming a membrane diffusion barrier and sorting proteins that transit to cilia. This ciliogenesis pathway is slightly distinct from the one occurring in mammalian cells, in which cilia assembly starts with centriole to BB maturation, migration to the cell surface and docking to the plasma membrane with the axonemal elongation starting either intracellularly or once anchored at the cell surface (Sorokin, 1968).

*Paramecium* BBs are organized into parallel longitudinal rows and into fields. This precise BB and cilia organization at the cell surface allows the cell to swim, feed and mate (Iftode et al., 1989; Tassin et al., 2015). The invariant field, located in the anterior part of the cell, shows cortical units with two ciliated BBs, while the posterior of the cell displays units with a single BB. In anterior or posterior cortical units, each BB, whether single or double, contains a cilium arising from a BB. In between, the mixed field exhibits units with either a single or two BBs (Tassin et al., 2015) (Figure 2B). The details of the organization of BBs are addressed in multiple figures below.

Basal bodies show microtubule triplets organized in a 9 fold symmetry, which is broken by the asymmetrical distribution of the three associated appendages, i. e, striated fiber (also called striated rootlet, SR), transverse microtubule and post-ciliary microtubules, all assigned to a specific microtubule doublet (Sperling et al., 1991) (Figures 2F,G). Therefore, BB rows together with the striated rootlets define the antero-posterior polarity as well as the left-right asymmetry of the *Paramecium*, which is critical for the organization of multiple BBs at the surface of this cell (discussed in (Nabi et al., 2019). The precise BB organization arrangement together with their ciliation pattern are responsible for the coordinated ciliary beating with pre-determined polarity (Iftode et al., 1989; Iftode & Fleury-Aubusson, 2003).

Since cilia are present in almost all groups of eukaryotes and their structural and molecular conservation throughout evolution, they are studied in a variety of species, giving us an understanding of their functions in various cellular environments. In humans, defects in ciliary function and formation lead to various pathologies called ciliopathies, which are complex multisystem human disorders affecting multiple organs as kidney, brain, eye, airways, and limbs, underlying numerous syndromes. Defective motile cilia in humans can lead to primary ciliary dyskinesia (PCD), characterized by compromised mucus clearance causing chronic airway diseases, defects in laterality, fertility, and brain development. Ciliopathies affecting non-motile cilia lead to several syndromes clinically diagnosed by the major organ(s) involved, and showing a spectrum of severity from relatively mild (Nephronophthisis (NPH), Senior-Loken, Bardet-Biedl (BBS)) to mid-severe to lethal (Joubert, Oral-facial-digital (OFD), and Meckel-Gruber Syndrome (MKS)) (Brown and Witman, 2014; Reiter and Leroux, 2017).

In the first part of this review, we will discuss *Paramecium* ciliary ion channels and other proteins (Table 1) that drive the ciliary beat form and frequency, which together control direction in swimming behavior. We will also address how cilia can be both motile and sensory (Bloodgood, 2010). We demonstrate the importance of multidisciplinary functional genomic approaches, in particular, the combined use of proteomics, genomics, fluorescent-epitope tagged expressed proteins, and electrophysiology with RNA interference (RNAi). In addition we demonstrate that the ciliopathy BBS gene products and Meckelin family of proteins have important roles in the formation of *Paramecium* cilia.

**TABLE 1:** Summary of the ciliary ion channels in this review organized by their membrane location, how they are activated, how they are trafficked to the cilia, and other pertinent characteristics including how they relate to ciliopathies.

*Paramecium* ion channels discussed in this review					
Gene	Location	Activation	Trafficking to cilia	Other characteristics	Relation to ciliopathies
Ca_V 1a,1b,1c_	Cilia only	Voltage dependent, depolarization	No dependence on BBS	Require Pawn proteins to be incorporated into the cilia	Essential in ciliary beating, therefore, informative for PCD
K_Ca_	Probably only in cilia	Calcium/Calmodulin and depolarization	BBS 1,3,4,7,8,9	Sk1a is the specific channel that we followed	Essential in ciliary beating, therefore, informative for PCD′ BBS cargoes
K_V_	Probably only in cilia	Voltage dependence	BBS 3,5		Essential in ciliary beating, therefore, informative for PCD; BBS cargoes
PKD2	Cilia and plasma membrane		BBS 7,8,9	No dependence on partner protein XNT to reach cilia	Provides insights into ADPKD; conductances of PKD2 and potential partners; BBS cargoes

In the second part, we will highlight recent work on high-resolution BB structure that enlightens us about how BBs resist various forces. Finally we use *Paramecium* as a model to 1) find insights in the mechanisms of BB anchoring and their critical positioning; 2) validate candidate genes for ciliopathies, in particular PCD.

### Voltage Gated Ca^2+^ Channels and the Action Potential

The extremely important observations of swimming behavior by Jennings (1906) piqued the interests of very talented physiologists, Eckert, Machemer, Naitoh, Kaneko and later others, who used electrophysiological approaches to show that motion of *P. caudatum* is controlled by ion conductances (Machemer, 1988a; Machemer, 1998b; Machemer, 1989). Hence, *Paramecium* became known as a little swimming neuron (Kung and Saimi, 1982). Frequency of the ciliary beat controls the swimming speed; the direction of the power stroke controls whether the cell swims forward or in reverse; and a turn results from the transition between forward and reverse swimming in the avoiding reaction. All of this motor behavior is under bioelectrical control in which channels of the cilia play key roles.

**FIGURE 3:**
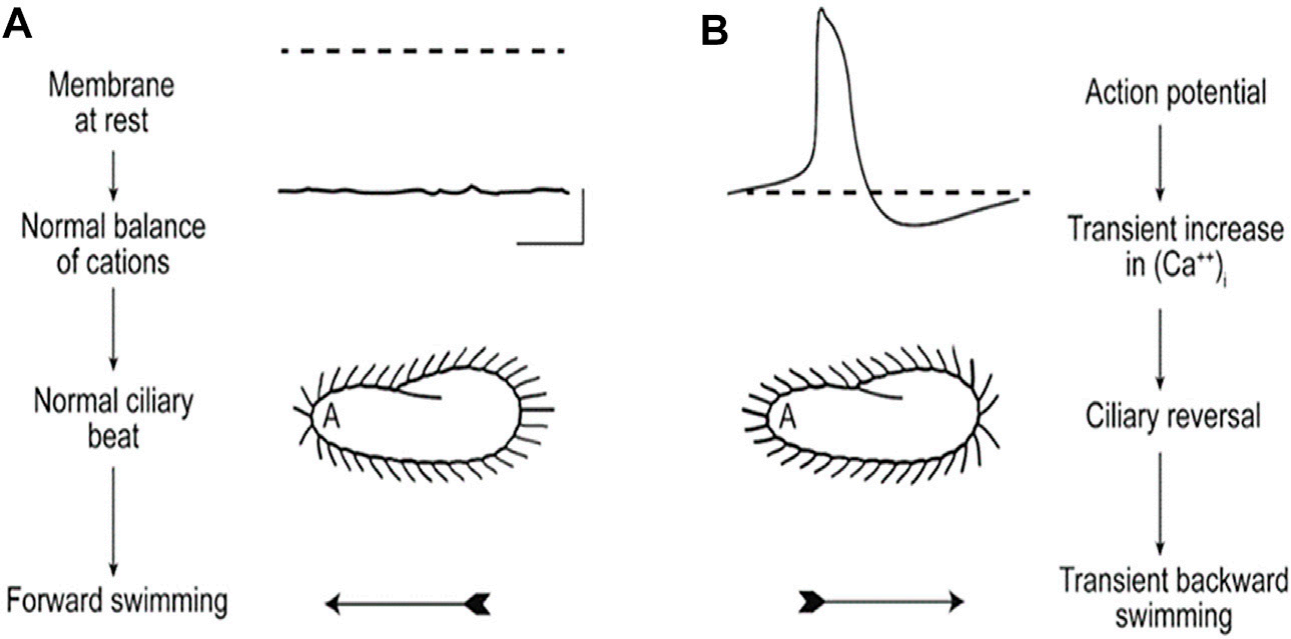
These images illustrate **(A)** that the resting membrane potential of *Paramecium* is negative; the ciliary beat is toward the posterior and cell swims forward. **(B)** In depolarizing conditions, such as high K or Ba solutions, the cell’s membrane depolarizes and reaches threshold for the action potential, during which Ca^2+^ enters the cilium through Ca_V_ channels and the Ca^2+^ changes the power stroke toward the anterior, moving the cell backward. The action potential is quickly terminated, returned to resting V_m_ levels, and the extra Ca^2+^ removed (Kung et al., 1975).

The speed of swimming is dependent upon the resting membrane potential that controls ciliary beat frequency (Brehm and Eckert, 1978a; Brehm and Eckert, 1978b; Kutomi et al., 2012; Machemer, 1988a; Machemer, 1998b; Machemer, 1989). Small hyperpolarizing stimuli increase beat frequency and swimming speed, while small depolarizing stimuli do the opposite. Depolarization beyond a threshold triggers the graded Ca^2+^ action potential by opening Ca^2+^ channels that are exclusively in the cilia (Dunlap, 1977; Machemer & Ogura, 1979). The increased intraciliary Ca^2+^ reverses the power stroke of the cilia toward the anterior, causing the transient backward swimming. The membrane potential is returned to resting levels by a rapid voltage activated K^+^ conductance and a slower Ca^2+^ activated K^+^ conductance, which responds to the Ca^2+^ coming into the cilium through the voltage gated Ca^2+^ channels (Satow & Kung, 1980). Like the voltage gated Ca^2+^ channels, these two types of K channels might also be concentrated in the cilia and absent from the Soma (Brehm & Eckert, 1978b). This very short summary of painstaking work on the underlying physiology of the ciliary beating and swimming behavior of *Paramecium* is captured in Figure 3 (Kung et al., 1975).

Kung had the insight to isolate behavioral mutants of *Paramecium* as an approach to identifying the channel proteins that regulate the action potential and swimming behavior. The resulting Pawn mutants, which were named for the chess piece that can move only forward, cannot back up if they encounter an obstacle or encounter depolarizing stimuli that open the Ca_V_ channels and should initiate an action potential (Kung et al., 1975). In addition to the three Pawn mutants, deciliation of the cells provided a way to study conductances in the absence of *ciliary* conductances (Machemer & Ogura, 1979). Deciliated cells survive and regrow their cilia even while impaled by microelectrodes. This allowed Dunlap to show that there were Ca_V_ channels specific to the cilia for the calcium action potential and that Soma channels did not contribute. As she watched the cilia re-grow, she could see that action potentials returned after partial regrowth of the ciliary length, suggesting that Ca_V_ channels were not evenly distributed and more prevalent at the distal end of the cilia (Dunlap 1977).

Despite the identification and characterization of the Pawn mutants 50 years ago, the Pawn gene sequences were not immediately evident. At that time, the use of *Paramecium* as a model organism was slowed by the sparsity of molecular tools and lack of an annotated genome. Nonetheless, through demanding techniques like complementation cloning, the Kung group identified the sequences coding for Pawn A and B at the turn of this century (Haynes et al., 1998; Haynes et al., 2000). These genes code for small proteins which could not be the major alpha 1 subunit of a Ca_V_ channel and are not like any of the auxiliary subunits of these channels known in other organisms.

It took until 2016 to reveal the genes and express the proteins of the very large (>250 kDa) Ca_V_ channels specific to the *Paramecium* cilia. The critical annotation of the *Paramecium tetraurelia* genome did not immediately hold all the answers about which apparent channel genes coded for cilia-specific channels because three whole genome duplications in its past made the sorting through many paralogs and ohnologs challenging (Aury et al., 2006). We employed LC-MS/MS analysis of the whole cilia and ciliary membrane (Yano et al., 2013) to help us identify which among the many Ca_V_ candidate channels in the *Paramecium* genome were specific to the cilia and found three Ca_V_ channel alpha subunits. Epitope tagging and expression of these very large proteins was challenging but allowed us to confirm their presence specifically in ciliary membranes. With RNAi we confirmed that these three identified in the ciliary membrane Ca_V_ channel alpha subunits participated in the calcium action potential (Lodh et al., 2016).

Identification of these cilia-specific Ca_V_ channels allowed us to answer the question why Pawn mutants swim only forward. It was known since 1973 that if Ca^2+^ could reach the axoneme of permeabilized Pawn mutants, the cilia could physically reverse beat. Presumably in live, intact Pawn cells, Ca^2+^ does not enter the cilia through channels to reverse the ciliary power stroke (Kung and Naitoh, 1973). The defect in intact Pawn cells could be due to non-functioning Ca_V_ channels in the Pawn cilia or failure of the channels to locate in the cilia. We ultimately found that in order for the Ca_V_ channels to traffic to and function in the cilia, the cells must have functional Pawn A and B proteins. In the absence of these proteins, the Ca_V_ channel alpha 1 subunit proteins are not located in the cilia (Lodh et al., 2016).

### Potassium Channels of the Cilia: Trafficking to the Cilia

The end of the action potential and repolarization of the membrane potential to rest is accomplished by Ca^2+^ feeding back to inactivate the Ca_V_ channel and activation of two types of hyperpolarizing K channels (Brehm & Eckert, 1978a). The voltage dependent ciliary K channel (K_V_) is activated by the depolarization phase of the action potential and the slower calcium-dependent ciliary K channel (K_Ca_) is activated by the Ca^2+^ that enters the cilium through the Ca_V_ channels during the action potential (Satow & Kung, 1980; Saimi et al., 1983). The physiological characteristics of these channels and more were known for many years (Kung and Saimi, 1982), but identification of their genes and proteins lagged behind. Once the first K gene was identified, additional K channel genes were found to be overwhelmingly abundant (perhaps 800 or more) (Haynes et al., 2003), making the search for ciliary K channels that are activated by the action potential challenging. In addition, the high similarity in gene sequences makes it almost impossible to silence a single gene because of off-target effects and difficult to silence a whole paralog group of K channel genes without affecting others.

**FIGURE 4:**
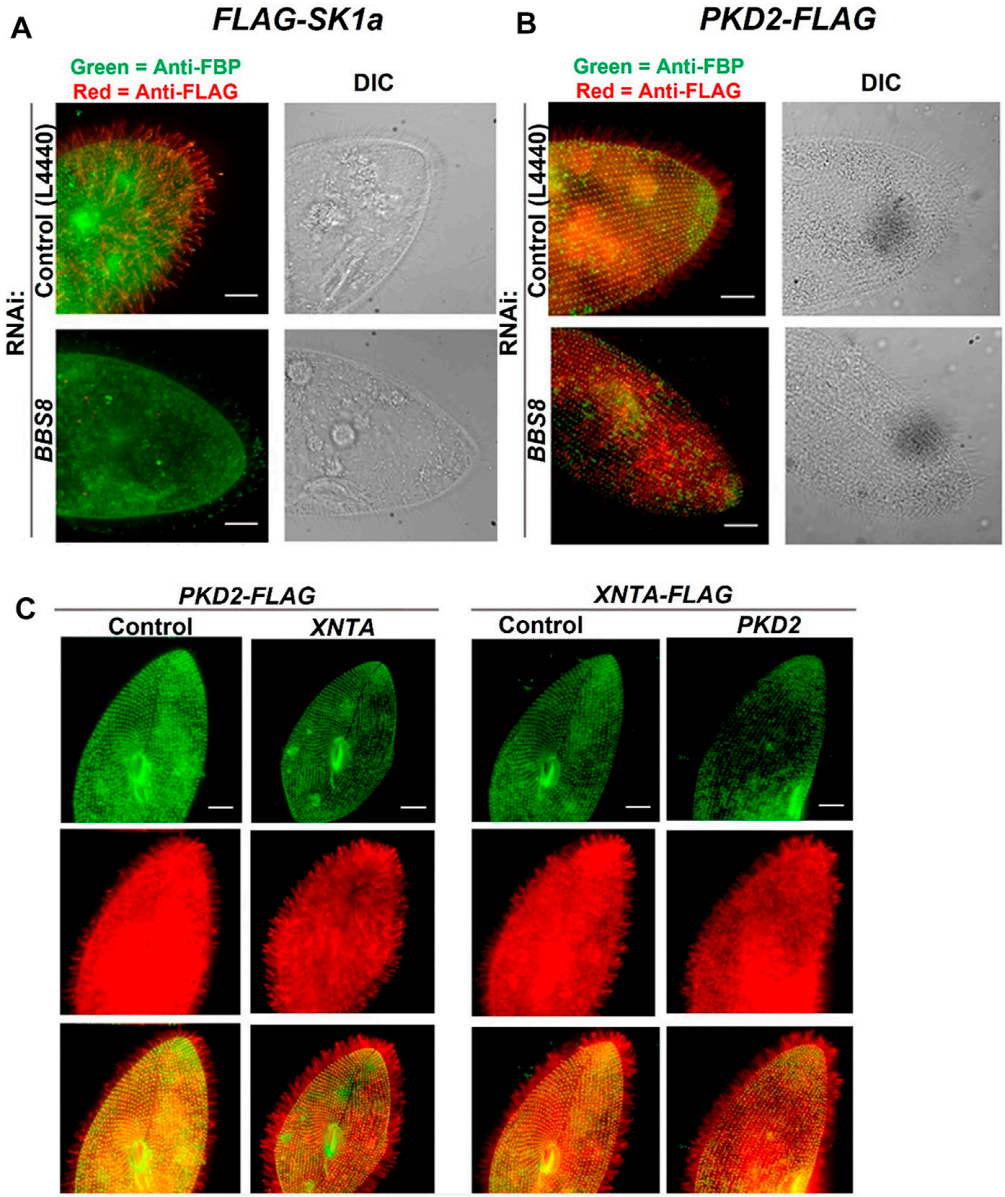
Immunofluorescence and RNAi allow us to follow the location of channels and other proteins in the cell. **(A)** immunofluorescence allowed us to localize the Sk1a channel (FLAG-Sk1a, red) to mainly the cilia of *Paramecium* (green staining shows the GPI-anchored folate binding protein, FBP). Upon feeding *BBS8* RNAi, the *FLAG-SK1a* disappears from the cilia, showing the dependence of this channel on the BBSome for trafficking. The GPI-anchored FBP remains undisturbed. Similarly **(B)** cells expressing *PKD2-FLAG* show the Pkd2 channel at the cell surface and in the cilia (red; basal bodies are green, stained with anti-*Tetrahymena* centrin) and after feeding the cells RNAi bacteria to deplete *BBS8*, the *PKD2-FLAG* channel is absent from the cilia. We have also used expressing cells and RNAi to examine the dependence of interacting proteins on one another for trafficking **(C)** Cells expressing *PKD2-FLAG* depleted in *XNTA* show no change in the localization of the Pkd2 channel and visa versa; cells expressing *XNTA-FLAG* depleted in *PKD2* show no change in the localization of the XntA protein. Scale bars represent 15 µm **(A)** and **(B)** from Valentine et al., 2012 reproduced with changes with permission using Creative Commons CC BY license **(C)** reproduced with permissions from Valentine et al., 2019 with permission using Creative Commons CC BY license.

In more recent studies, we used LC-MS/MS to identify K_Ca_ channels from the ciliary membrane (Yano et al., 2013). When epitope tagged, one of these channels (SK1a, Figure 4A) could be visualized in the cilia but appears to be absent from or much less abundant in the cell body membrane (Valentine et al., 2012).

To reach their location in the cilia, these channels rely upon the Bardet Biedl Syndrome complex (BBS or BBSome) that is thought to act as a coat in trafficking select proteins to (or retaining them in) the cilia (Jin et al., 2010). The BBS proteins are required for trafficking of G Protein Coupled Receptors to and olfactory cilia ion channels (Berbari et al., 2008; Uytingco et al., 2019). We knew from behavioral tests for long backward swimming after stimulation that cells with their *BBS 1,4,7,8 or 9* genes silenced lost K_Ca_ channel function; those with their *BBS 5* gene silenced lost the function of K_V_ channels of the cilia; those with *BBS 3* silenced lost function of both kinds of K channels. Therefore, both types of ciliary K channels rely upon the BBSome for trafficking. Interestingly, not all channels of the cilia depend upon the BBSome. The Ca_V_ channels do not require the BBSome in *Paramecium* to reach the cilia, but another channel (PKD2) does (Figure 4B) (Valentine et al., 2012) as we discuss below.

Figure 4 reinforces the usefulness of functional genomics: epitope tagged expressed proteins (hence the red and green fluorescence) combined with RNAi show location and dependence upon BBS; the channel proteins were identified by cilia proteomics and their genes by genomics. These functional genomic techniques are especially useful in concentrating and studying low abundance channels, such as Cav ciliary channels (Lodh et al., 2016).

### After the Action Potential

As described above, the membrane potential returns to rest quickly after the action potential due to hyperpolarizing K conductances, but reduction of ciliary calcium levels back to base levels lags behind, making the duration of backward movement longer than the duration of the action potential. Calcium must be removed from or sequestered in the cilia for at least two reasons. Calcium feeds back on the voltage gated Ca channels of the cilia and inactivates them. To respond to the next depolarization, this Ca^2+^ inactivation must be relieved. Secondly, the reverse power stroke of the cilium is in response to the Ca^2+^ that enters through these channels to interact with the axonemal proteins. To resume forward swimming, Ca^2+^ at the axoneme must be removed. Below we use the duration of backward swimming as a read-out of the calcium remaining in the cilium following the action potential.

The mechanism by which Ca^2+^ is removed from the vicinity of the axoneme and the Ca_V_ channels has been discussed for many years. The Ca^2+^ binding protein calmodulin has been suggested to capture this ciliary Ca^2+^, but there are other considerations. Reduction of calmodulin levels in *Paramecium* can be achieved through electroporation of calmodulin antisense oligonucleotides (Fraga et al., 1998). However, these electroporated cells do not extend their backward swimming episodes in depolarizing solutions as would be expected if calmodulin were responsible for sequestration of the Ca^2+^ from the action potential (Fraga et al., 1998; Yano et al., 1996). Complicating these results is the calmodulin dependent Na_Ca,V_ channel that would also be inhibited, but its inhibition causes the opposite behavior of reduced backward swimming. The bottom line is that, with reduced calmodulin, the duration of backward swimming is not prolonged but its root cause needs more examination. Also arguing against calmodulin as the major mechanism for removing Ca^2+^ from the cilium are the *Paramecium* mutants with abnormal C or N terminal lobes of calmodulin. Their behavioral changes are not consistent with failure to remove Ca^2+^ from the interior of the cilium (Kink et al., 1990; Kung et al., 1992; Preston et al., 1991). Centrin, another EF hand protein, rescues a *P. caudatum cnr* mutant, but this also is not consistent with a role for centrin in sequestering ciliary Ca^2+^ from the channel (Gonda et al., 2004; Gonda et al., 2007).

With the advent of more modern molecular techniques, another mechanism has gained support for the rapid removal of Ca^2+^ from the cilia: transport through plasma membrane calcium ATPase pumps (PMCAs) (These pumps are not limited to the plasma membrane, but, as you will see, are in the ciliary membrane as well). There are many (23) genes for PMCAs in the *Paramecium* genome, but only a small subset of their proteins are found in the cilia (Yano et al., 2013). Two of these pumps (ptPMCA2a and 2b) appear to play a role in controlling the levels of Ca^2+^ following the action potential; RNAi for only these two very specifically prolong the duration of backward swimming. These two pumps are also found to co-immunoprecipitate and fractionate in the same ciliary lipid fractions with the Ca_V_ alpha 1 subunits of the calcium channels that are specific, i.e. exclusively in, to the cilia (Yano et al., 2015; Yano et al., 2021). These results suggest that these Ca^2+^ pumps are in proximity with the channel, where they are well positioned to relieve Ca^2+^ feedback and inhibition of the channel and prepare the channel for the next round of excitation.

### PKD2 Channels in the Cilia: More Insights Into Trafficking

The polycystic kidney disease channel (PKD2) is a non-selective cation channel in mammals (Delmas et al., 2004; González-Perrett et al., 2001). Inhumans, dysfunction of PKD2 or its most common interacting partner PKD1 leads to nearly all cases of Autosomal Dominant Polycystic Kidney disease (ADPKD) (Boucher and Sanford, 2004). PKD2 has a homolog in *Paramecium* that seems to collaborate with a protein known for Mg induced behavior, XntA (Eccentric A) that resembles the partner for PKD2 in higher organisms (PKD1) (Haynes et al., 2002; Preston & Kung, 1994; Valentine et al., 2019)*.* Both of these proteins, Pkd2 and XntA, are found in cilia and cell body membranes, but neither depends upon the other for trafficking to the cilia (Figure 4C). By following proteins from tagged expression vectors, we see that RNAi depletion of Pkd2 does not affect the localization of XNT in the cilia and vice versa. The results of a multidisciplinary study including electrophysiology, RNAi, behavioral analysis and more show that Pkd2 functions as a Mg^2+^ permeable channel while in the cell body and cilia (Valentine et al., 2019). While optimal permeability of the cell and behavioral response of the cell to Mg^2+^ occurs when both Pkd2 and XntA are present, it is the Pkd2 protein that is the Mg^2+^ channel. Pkd2 depends upon the BBSome to traffic to the cilia, but XntA does not.

The story of these two proteins, XntA and Pkd2, illustrates the tale of two membranes, which we describe in more detail later. The different contexts of the ciliary and cell body membranes are important for the function of the Pkd2 channel (Valentine et al., 2019). Both XntA and Pkd2 are present and potentially interact in both membranes. However, while Pkd2 can function alone in the cell membrane, e.g. in decliated cells, the presence of cilia inhibits the Pkd2 activity.

The story of *Paramecium* Pkd2 and XntA shows that new insights can be gained into these ciliopathy proteins for their conductances and their potential interacting partners.

### Other Stimuli: A Tale of Two Membranes

One goal of this review is to describe the ion channels of the *Paramecium* cilium that are responsible for the bioelectric control of the frequency and shape of the ciliary beat (Eckert, 1972; Eckert and Naitoh, 1972). The Ca_V_ and K channels discussed so far apparently are exclusive to the cilia, but they can be affected by channel or second messenger activity generated at the cell body plasma membrane. The cell is isopotential, meaning that whatever electrical activity there is present in the plasma membrane, is immediately detected across the entire cell. For example, a depolarizing mechanosensory receptor potential of the plasma membrane generated by a tap on the anterior of the cell by a predator can open ciliary voltage gated channels and initiate the action potential. We discuss some examples of this tale of two membranes, ciliary and plasma membrane, which are contiguous but have different lipid and protein complements.

A touch on the cell’s anterior triggers a depolarizing receptor potential, and, if this potential is large enough, the ciliary Ca_V_ channels open with the action potential and avoiding reaction as seen in Figure 2. In contrast, a touch on the posterior causes the cell to swim fast forward due to hyperpolarizing K channels (Machemer, 1974; Machemer, 1988a). The receptor potentials for mechano-stimulation can be seen in deciliated cells, meaning that the cilia are not needed for this aspect of mechanoreception. The cilia are, however, necessary for the motor response of an avoiding reaction or fast escape swimming. Hence, the term “tale of two membranes” refers to the role that the cell surface membrane and its channels and receptors play in ultimately affecting the channels of the cilia that control beat and frequency.

Chemical stimuli affect swimming behavior. In general, chemical stimuli are grouped into attractants that signal food (bacteria) to the paramecia and repellents that generally signal toxic conditions, like extremes of pH or salt (Van Houten, 1992; Van Houten and Preston, 1988). Many attractants are bacterial metabolites including acetate, folate, biotin, glutamate, extracellular cyclic AMP and ammonia (Davis et al., 1998; Bell et al., 2007). All but ammonia have measurable binding sites on the cell, presumably on receptors. The exception is ammonia, which diffuses across the membrane and alkalinizes the cell, resulting in hyperpolarization and altered swimming (Davis et al., 1998).

The result of application of any of these attractants to the cell is an immediate change in swimming: decrease in frequency of turning and increased swimming speed, which results in congregation of the cells in the area of attractant by a biased random walk (a kinesis mechanism) (Preston and Van Houten, 1987; Van Houten et al., 1982; Van Houten, 1979, Van Houten et al., 2000; Van Houten and Preston, 1988). Repellents increase the frequency of turns due to action potentials and slowed swimming speed, causing dispersal also by a biased random walk. The reader will infer from the preceding discussion of *Paramecium* physiology and behavior that cells hyperpolarize in attractants and depolarize in repellents (Van Houten, 1994; Van Houten and Preston, 1988).

Observations of cells’ swimming behavior is often done using micropipets to transfer cells one by one into a new solution. The behaviors of cells as they are immersed into pools of attractant or repellent are informative about their inner physiological changes. However, cells in pond water swim into and out of areas of stimulus. Their behavior as the cell anterior first juts across a step gradient into attractant (called an on response) is also characteristic of hyperpolarization. The on-response hyperpolarization is typical of a K^+^ conductance, like the transient K^+^ conductance that was identified by stimulation of cells with L-glutamate (Preston & Usherwood, 1988). However, response to other attractants can be different. As long as these attractants continue to be present, the on-response hyperpolarization is sustained, which we attribute to an electrogenic calcium ATPase pump conductance (reviewed in (Van Houten, 1998)). Movement of a cell out of the attractant (called the off response) is characterized by an immediate turn and reduction in speed of swimming, resulting from a depolarization sufficient to open ciliary Ca^2+^ channels and decrease ciliary beat (Nakaoka and Machemer, 1990). Previously we had shown through computer simulation that the immediate response of the cell upon entering the area of attractant acetate is critical for attraction of the population; even more critical than the immediate turn upon leaving the area of acetate (Houten and Van Houten, 1982).

In order to identify the ion channels that participate in the on and off responses of chemical stimuli, we chose two attractants (acetate and biotin) and employed a selection of behavioral mutants with known conductance defects (Kink et al., 1990; Ling et al., 1994; Preston & Kung, 1994). The outcome was that the most important behavior for the successful attraction to acetate was the on response, as our computer simulation had long ago suggested. Correlating the known conductance defects of the mutants with their defects in chemoresponse allowed us to assign the on-response of the attractant biotin to a conductance dependent upon the *I*_K(Ca,h or d)_ and the off response to a Ca conductance (*I*_Ca_) that is large enough to open Ca^2+^ dependent channels responsible for conductances *I*_K(Ca,d)_, *I*_Na(Ca)_ and *I*_Mg(Ca)_ (Bell et al., 2007). Except for the (*I*_Ca_) and *I*_K(Ca,d)_, which may be the ciliary channels that we described above, we have not identified the proteins responsible for these conductances. The *I*_Mg(Ca)_ may be equivalent to the XntA Mg channel partner that is both in the cilia and cell body membrane (Valentine et al., 2019).

Cilia have an array of binding sites for chemical stimuli. L-glutamate binds to cilia as a ligand (Preston and Usherwood, 1988). We have subsequently found a candidate for the L-glutamate receptor on the cilia using MS and other analyses (Romanovitch, 2012). The L-glutamate receptor appears to be homologous to an NMDA-like protein (Romanovitch, 2012). Preston and Usherwood measured binding to ciliary membranes and also noted that deciliated cells hyperpolarize in L-glutamate. Judging from these results, we expect that the L-glutamate receptor should be present on both the cilia and cell body membranes (Preston & Usherwood, 1988), which our biochemical and proteomic studies have confirmed (Romanovitch, 2012).

The receptors for other attractants such as folate can be found on the cilia and cell body (Schulz et al., 1984; Valentine et al., 2012; Weeraratne, 2007). The signal transduction mechanisms that allow this peripheral glycosylphosphatidyl inositol (GPI) anchored protein receptor to initiate hyperpolarization of the membrane are not understood. Possibly it is achieved through interactions with other proteins of lipid rafts in the cilia, where GPI anchored proteins could concentrate along with ion channels.

We have known for a long time that the attractant *extracellular* cyclic AMP binds to whole cells (Smith et al., 1987). We now know by using the techniques of RNAi, LC-MS/MS and genomic sequence analysis that there are two receptors (pCAR 1 and 3) related to the *Dictyostelium* cAMP G Protein Coupled Receptors (Czapla, 2012; Firtel et al., 1989; Franca-Koh et al., 2006; Schaap, 2016; VanHaastert and Devreotes, 2004; Willard and Devreotes, 2006) that function to mediate attraction to cAMP (Czapla, 2012). These *Paramecium* receptors (pCAR1 and 3) are found in the cell body membrane and pCAR1 alone is found in the cilia (Czapla, 2012).

It remains to be seen how the binding of stimuli to these receptors on cell body and cilia is transduced into a hyperpolarization of the cell. The on and off responses originate with the stimuli as they bind to and then diffuse away from their receptors, but we do not yet know how receptor occupancy is transduced into ion channel activity.

While we focus on these ciliary channels and a few plasma membrane channels, it is important to remind ourselves that the cell has other ion currents characterized by voltage clamp. Their channels are located in the Soma membrane or cilia, or both (Kung & Saimi, 1982), but their channel proteins are not known.

### Modulation of Ciliary Beating by Intra-ciliary Cyclic AMP

*Paramecium* can be treated as a neuron and its membrane potential manipulated by concentrations of extracellular ions. Lowering extracellular K concentration hyperpolarizes the cells, which induces the formation of the second messenger, cyclic AMP, in the cilia and cell body (Schultz et al., 1992; Schultz et al., 1997; Weber et al., 2004). The cyclic AMP in the cilia, in turn, activates protein kinase A, which modifies the axonemal dyneins, resulting in increased beat frequency, changed beat shape, and faster forward swimming (Hamasaki et al., 1991; Satir et al., 1993; Satir et al., 2014).

Although the chemical stimuli discussed above hyperpolarize the cells, only the stimulus L-glutamate elicits the second messenger, cAMP. When we hyperpolarize the paramecia by dramatically reducing extracellular K, the cells swim fast and smoothly and increase intracellular cAMP (Bonini and Nelson, 1988; Bonini and Nelson, 1990; Bonini et al., 1986; Schultz et al., 1992; Yang et al., 1997). Remarkably, the small hyperpolarization induced by glutamate induces an extremely rapid (< 30 msec) increase in intracellular cAMP (Yang et al., 1997). Thus, it appears that not all hyperpolarizing potential changes resulting from stimuli are interpreted alike by the cell because other chemical stimuli that elicit similar size hyperpolarizations do not increase cAMP and must accelerate ciliary beating by other means (Yang et al., 1997).

The glutamate story has other interesting aspects. Just as in umami (glutamate) taste, L-glutamate synergizes with 5′GMP, while D-glutamate also is an attractant that acts independently of L-glutamate (Van Houten et al., 2000).

An interesting link between the hyperpolarizing K^+^ conductances and increased cyclic AMP production came from the finding by Schultz that a cyclase and K channel were domains of the same fusion protein (Weber et al., 2004). The adenylyl cyclase-K channel proteins are found both in the cell body and in the cilia (Weber et al., 2004; Yano et al., 2013). However, these K channels do not participate in the repolarization after the action potential.

Understanding the formation and function of *Paramecium* cilia can provide insights into mammalian cilia and ciliopathies as well (Valentine and Van Houten, 2021; Valentine et al., 2019). In particular, ion channels of the *Paramecium* cilia show conserved structure and function with mammalian channels, and can provide some advantages: Paramecia are amenable to electrophysiological studies, forward and reverse genetics, gene silencing, cutting edge microscopy techniques, and harvesting of a large amounts of material for biochemical and proteomics studies.

### Swimming Behavior Highlights Aberrant Ciliary Activity or Formation

For efficient swimming the cell needs more than beating cilia. The cilia must be organized into rows relative to the poles of the cell, with proper rotational orientation of basal bodies and at a critical distance apart for metachronal waves to form for efficient swimming. These requirements are in common with metachrony in multi-ciliated metazoan cells (Brooks and Wallingford, 2014; Spassky and Meunier, 2017). As shown in Figures 2A,B, BBs in *Paramecium* are arranged in longitudinal rows, and their polarity is marked by the asymmetrical organization of their associated appendages. One of these appendages, the striated rootlet (SR), attaches to specific microtubules in the BB and is composed in large part with SF-assemblin (Nabi et al., 2019).

**FIGURE 5:**
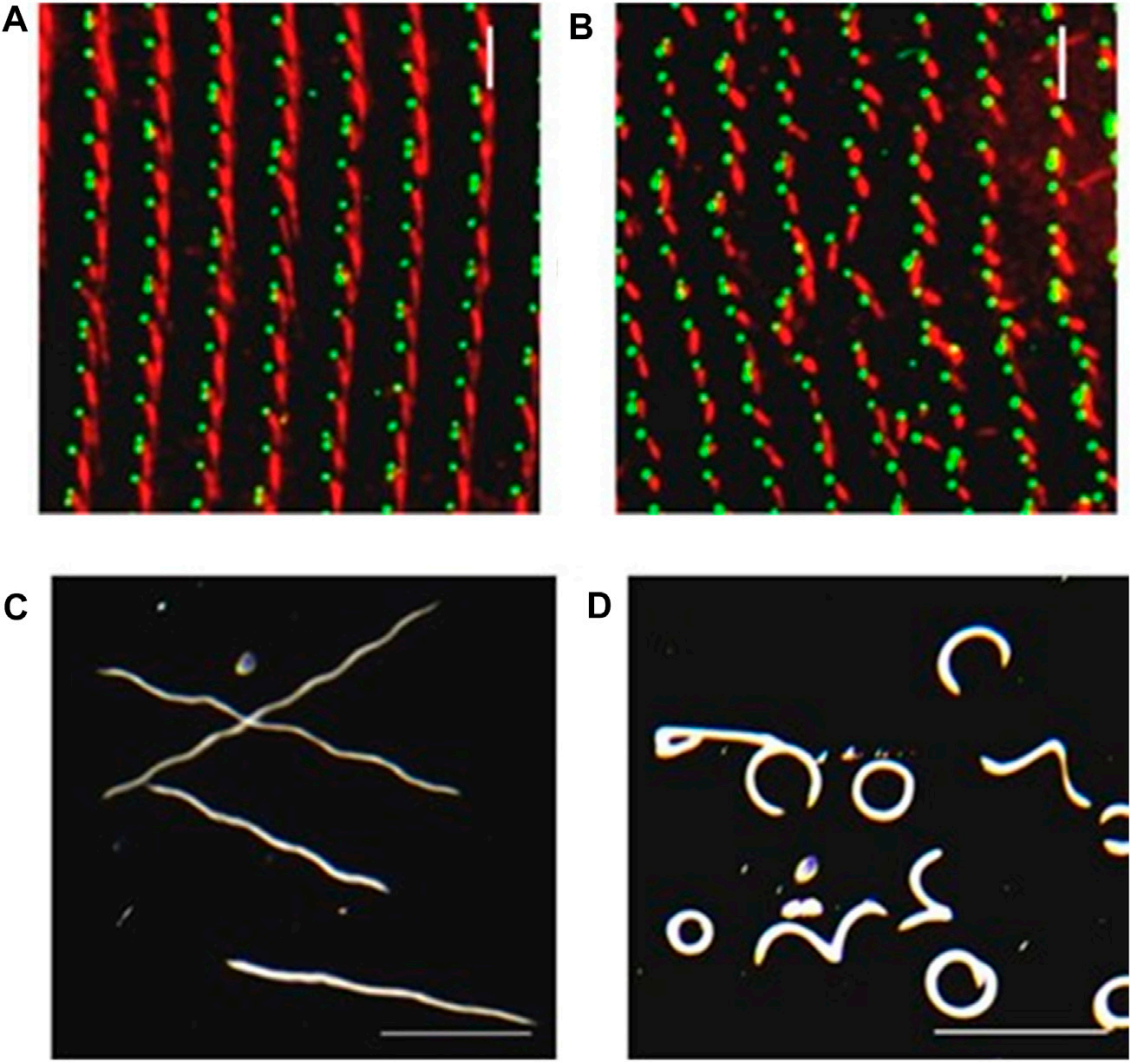
Depletion of SR gene products can lead to basal body and row misalignment. **(A)** Control cell (left) and RNAi treated cell with reduced SR proteins. **(B)** with basal bodies identified in green with ID5 antibodies, and the SRs identified in red with anti-SR antibody. Note misalignment of basal body rows and mis-orientation of basal bodies. **(C)** Control cells and **(D)** RNAi treated cells with reduced SR proteins as they swim in a pool of resting buffer for the same period of time. Note that the mis-orientation of basal bodies and rows leads to abnormal swimming (Nabi et al., 2019).

For instance, after disruption of the SR with RNA interference (RNAi), the cilium can lose orientation (Figures 5A,B (Nabi et al., 2019),). Instead of all oars rowing in the same direction, the cilia act like oars pulling in random directions (Nabi et al., 2019), sending the cell into circles or other departures from straight swimming. Figure 5C-D shows the inability of cells with disrupted Striated Rootlets to swim efficiently and in a straight forward track.

Similarly, disruption of human ciliopathy genes by RNAi in *Paramecium* cause erratic swimming. The Bardet Biedl Syndrome proteins participate in the trafficking of proteins, including some channels, to their location in the cilia (Valentine et al., 2012; Picariello et al., 2014). When ion channels do not reach their proper location in the ciliary membrane, ciliary beating and behavior are affected as described above. However, reduction of some of the BBS proteins also leads to loss of cilia causing slow swimming and as well as abnormal swimming patterns.

Meckelin family ciliopathy proteins are necessary for the proper localization and formation of cilia (Hartill et al., 2017). As in other systems, reduction of MKS3 leads to short and missing cilia in *Paramecium* and also a new phenotype of misalignment of longitudinal rows of basal bodies, rotation of the orientation of the basal bodies and their rootlets (Picariello et al., 2014). Loss of cilia would cause irregular movement of the cilia and loss of metachrony. The number of cilia is also reduced by RNAi for MKS1 leading to abnormal swimming patterns (Funfak et al., 2015). While these abnormalities are due to orientation and structural problems and not due to the bioelectric control of the cilia, nonetheless they lead to highly disrupted swimming. MKS5 in contrast, when depleted, causes loss of cilia and slow swimming, but has no effect on straight basal body rows or orientation of rootlets (Valentine and Van Houten, 2021).

The functional analyses of genes involved in *Paramecium* ciliary function following RNAi knockdowns of candidate genes, for example, are expedited by the easy detection of changes in swimming behavior. Interesting prospects can then be followed up with gene overexpression, electron microscopy observations, and protein localization by IF, all of which are rapid, efficient and reproducible methods. These attributes of *Paramecium* contribute to its appeal as a model organism for the study of ciliogenesis.

### Insights in Basal Body Architecture Using Cryo-Electron Tomography

As discussed briefly above, BBs are spatially organized into parallel longitudinal rows and also into fields (Figures 2A,B). The invariant field, located in the anterior part of the cell, shows cortical units with two ciliated BBs, while the posterior part displays units with a single BB (Figure 2A1, A3, B). In these cortical units, each BB, whether single or double, contains a cilium arising from a BB. In between, the mixed field exhibits units with either a single or two BBs (Figure 2A2, Figure 2C). In the latter case, both BBs are anchored at the cell surface but only the posterior one is ciliated (Tassin et al., 2015).

Centriolar and BB structures are organized in three distinct parts: the proximal part containing both the cartwheel and the inter-microtubular linkage (the A-C linker), the central part containing the inner core, and the distal part (Figure 2). The latter corresponds to the transition zone and lies above the inner core containing part and below the cilium (Figure 2F). In the 1950s, transmission electron microscopy of resin-embedded samples from various species revealed the main centriolar ultrastructure. This led to the description of the 9-fold symmetry of the triplet. In the late 1960s, taking advantage of the large number of these structures found in ciliates, Dippell (Dippell, 1968) and Allen (Allen, 1969) deciphered the early steps of BB development in *Paramecium* and *Tetrahymena*, respectively. They propose that the nine-fold symmetry is due to the cartwheel (Dippell, 1968), which is the only structure required for centriole development (Allen, 1969). At the same time, Sorokin (Sorokin, 1968) described the centriole duplication process in mammalian multiciliated cells. Later on, using serial sectioning, Dute and Kung (Dute & Kung, 1978) described the organization of the *Paramecium* transition zone.

In contrast to resin-embedded samples, which require both dehydration and contrasting reagents such as use of heavy metal, cryo-electron microscopy (cryo-EM) preserves the hydrated state of the structure close-to-native condition; tomography requires the acquisition of two-dimensional (2D) projection images of the specimen in a range of orientations. The combination of both technics, cryo-tomography, allowed for the acquisition of a 3D volume at nm resolution of the structure after computational tomogram reconstruction from a tilt-series. However, cryo-tomography requires thin samples, about 100–300 nm thick, limiting direct *in situ* analysis, which requires a cryo-focused ion beam (FIB) (Schaffer et al., 2017). Using purified centrioles, this technique made it possible to decipher the assembly of the mammalian procentriole with a special focus on the microtubule triplet formation. The proximal end of the A-microtubule is capped by a gamma tubulin ring complex (γ-TuRC)-like structure and the B- and C-microtubules elongate bidirectionally from its wall (Guichard et al., 2012). To increase image resolution, sub-volumes of repetitive structures can be computationally extracted from tomograms, aligned, and averaged (sub-tomogram averaging). In addition, taking advantage of the 9-fold symmetry, a symmetrization of the data may be used to compensate for the missing wedge, a well-known artifact in cryo-tomography (Guichard et al., 2012). Using this strategy and capitalizing on the long and repetitive cartwheel observed in *Trichonympha*, Guichard et al. (Guichard et al., 2012; Guichard et al., 2013) resolved the complete architecture of the *Trichonympha* BB proximal region. The resulting 3D map reveals that the ring of the cartwheel central hub contains, in addition to the nine radial spokes, additional densities called Cartwheel Inner Densities (CIDs). Since the Sas6 protein has been shown to be the main component of the cartwheel and that nine homodimers of Sas6 assemble into a ring from which nine coiled-coil rods radiate outward, Kitagawa et al. and van Breugel *et al.* (Kitagawa et al., 2011; van Breugel et al., 2011) generated a TaSAS-6 (SAS-6 orthologous *Trichonympha agilis*) ring model, and demonstrated that it fits perfectly inside the observed ring densities of the cartwheel central hub.

**FIGURE 6:**
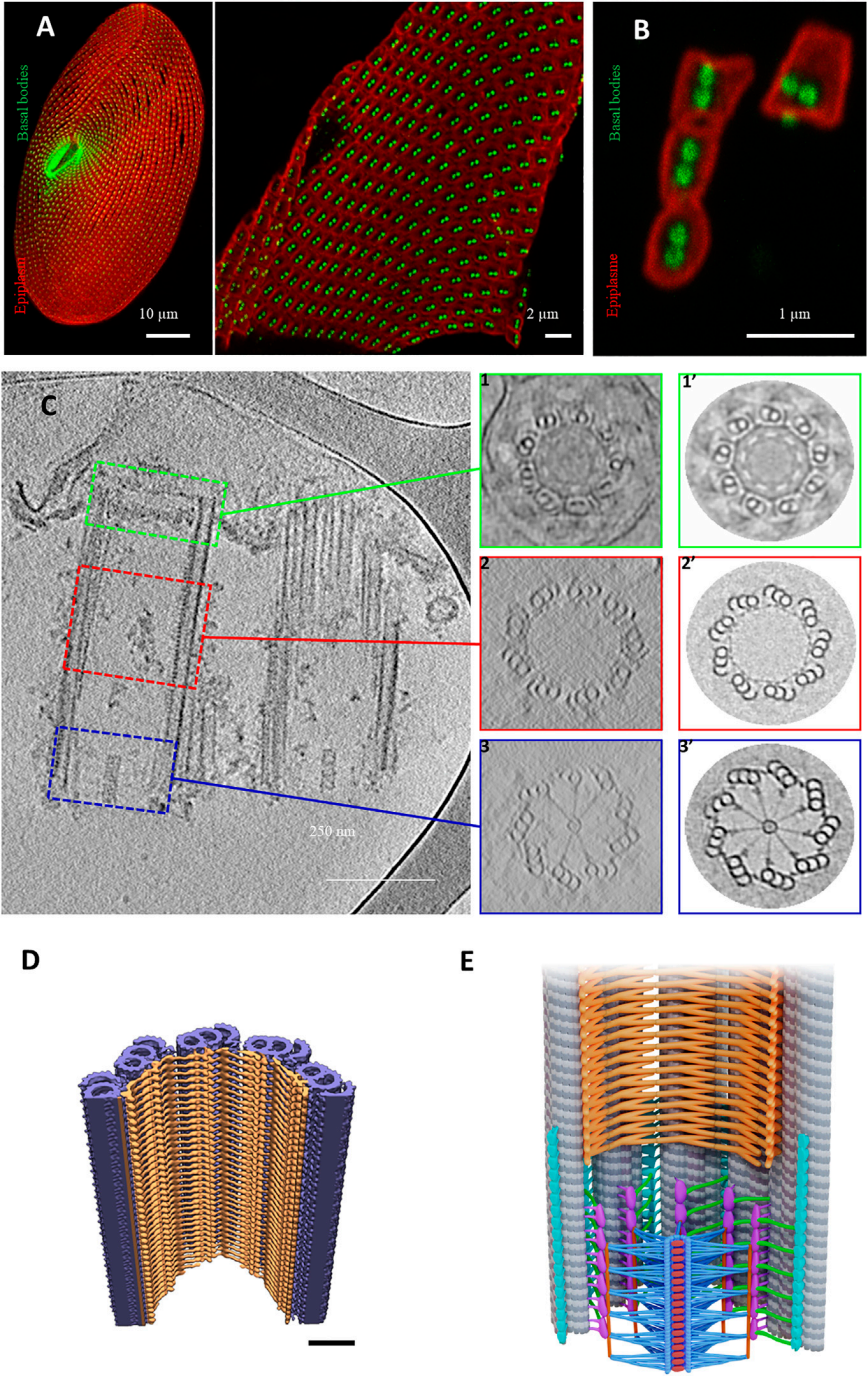
Basal body organization in *Paramecium tetraurelia*. **(A)** left: *Paramecium* stained Basal bodies (BB) are decorated by polyclonal anti-glutamylated tubulin (green) and anti-epiplasmin antibodies (red). Right: purified Paramecium cortex stained with anti-glutamylated tubulin (green) and anti-epiplasmin antibodies (red). Scale bar: 10 µm. **(B)** Purified basal body units obtained by 5 s of sonication. Scale bar: 2 µm. **(C)** Cryo-electron tomography of two *Paramecium* basal bodies. Z slice from a tomographic three-dimensional reconstruction showing a longitudinal section of a two basal body-cortical unit with the transition zone (green square), the central region (red square) and the proximal region (blue square). 1, 2, 3: Transverse projection of the cryo-tomogram at the respective three levels; 1′, 2′, 3’: 9-fold circularizations have been applied to the transverse projection. 1-1′, show the transition zone, 2-2′ show the central region of the basal body and 3-3′ show the basal body proximal region with the cartwheel structure inside. **(D)** The inner scaffold forms a dense helical lattice. Three-dimensional (3D) view of the ninefold symmetric central regions from *Paramecium* BB. Unrolled inner scaffold structures. In *Paramecium* a 2-start helix is observed (the pink color indicates one helix while the orange one indicated the second helix). Adapted from Le Guennec et al., 2020. **(E)** Cartoon of a longitudinal section of a Basal body. The microtubules are in grey, the cartwheel is in blue, CID in red, pinheads in violet, **(A–C)** linker in turquoise, triplet base in green and the inner scaffold in orange [Reprinted from (Klena et al., 2020; Le Guennec et al., 2020)]. The Authors, some rights reserved; exclusive licensee AAAS. Distributed under a CC BY-NC 4.0 License (http://creativecommons.org/licenses/by-nc/4.0/).

To examine whether the proximal end has been conserved throughout evolution, Klena et al. (Klena et al., 2020) analyzed this region by cryo-tomography on purified BBs from three species: *Paramecium tetraurelia*, *Naegleria gruberi* and mammalian cells. *Paramecium* cortex with its BBs can be easily purified, but it is too thick for cryo-tomography (Figure 6A). Therefore, we developed an approach to isolate cortical units from the cortex (Figure 6B); see Material and Methods in (Le Guennec et al., 2020). In addition to yielding a large number of cortical units, the *Paramecium* BBs in this preparation appear to be protected from compression in the ice layer, giving confidence in our analysis of the tomograms (Figure 6C). The results obtained on *Paramecium* BBs show that the cartwheel-containing region has a conserved organization when compared to that obtained from the *Chlamydomonas* centriole as observed *in situ* by cryo-focused ion beam (cryo-FIB) (Klena et al., 2020). Instead of being composed of a single ring as previously described, the central hub of these studied species is constructed of pairs of rings from which emanate the radial spokes (Klena et al., 2020). This result was also found in *Trichonympha* (Nazarov et al., 2020) suggesting a conserved feature of the cartwheel stacking. This novel 3D structure is proposed to fit with a model of two superimposed SAS-6 rings that are slightly offset (Nazarov et al., 2020). Inside the central hub, CIDs were observed in all species analyzed at the center of each ring-pair, suggesting that they might be important for their construction.

As previously mentioned, these structures are well conserved throughout evolution; however, some distinct features between species have been observed. For instance, the radial spokes are organized differently between *Paramecium*, *Chlamydomonas* and *Trichonympha*: in *Paramecium* and *Chlamydomonas,* each spoke is a combination of three substructures emanating from pair of rings, while in *Trichonympha* it is made of two substructures. This is probably due to the divergence of the Sas-6 coiled-coiled protein between these species. Altogether, data obtained using cryo-electron tomography suggest that 1) the cartwheel defines the polarity of the centriole; 2) the cartwheel, the triplet base and the A-C linker mainly achieve the cohesion of the centriole proximal part.

Le Guennec et al. (Le Guennec et al., 2020) were able to decipher the inner core region using *Paramecium* purified BBs and *in situ Chlamydomonas* BBs. Using subtomogram averaging of the microtubule triplet, the authors reconstructed the full inner scaffold from both species. Despite differences, the overall structure is well conserved and forms an extended helical meshwork that attaches to the microtubule triplet along the centriolar wall (Figures 6D,E). In *Paramecium*, the structure shows a two-start helix while in *Chlamydomonas* it contains a three-start helix. The presence of this complex meshwork structure suggests that it is required for microtubule wall integrity. Depletion of Centrin 2 by RNAi in *Paramecium* leads, in addition to BB internalization, to the disappearance of the internal structure of the microtubule wall of the duplicated BB together with a microtubule triplet loss (Ruiz et al., 2005). In mammalian cells, Centrin2 forms a complex with POC5, POC1B, FAM161A (Le Guennec et al., 2020) and WDR90 proteins (Steib et al., 2020). These proteins have been localized in U2OS mammalian cells by Ultrastructure Expansion Microscopy (U-ExM) (Gambarotto et al., 2019) to the centriole central part and excluded from both proximal and the utmost distal ends similar to Centrin 2, which also displays a distal end localization (Steib et al., 2020). Indeed, depletion of POC1B in *Tetrahymena* (Pearson et al., 2009; Meehl et al., 2016) and WDR90 in human cells (Steib et al., 2020) induce both a loss of BB stability and a missing microtubule triplet. In addition to its localization at the inner core region, a dynamic POC1B pool was also found in the cartwheel region as well as on the nascent BB (Pearson et al., 2009). Based on these results*,* Meehl et al. (Meehl et al., 2016) proposed a role for Poc1 for maintaining A-C linker integrity.

In addition to these inner core proteins, FGFR1 oncogenic partner (Fop1) functionally interacts in *Tetrahymena* with Bld10 and Poc1, which are required for BB stability (Bayless et al., 2016). Interestingly, Fop1 and also microtubule glutamylation have been asymmetrically enriched at triplet microtubules. The authors proposed that this asymmetric localization is necessary to stabilize BBs against the forces produced by ciliary beat (Bayless et al., 2016).

Taken together, the large quantity of purified cortical units obtained from paramecia makes it possible to use cutting-edge technologies to decipher the 3D ultrastructure of BBs at a resolution of 20–40Å. These results would provide more insights through comparisons with cryo-tomograms of human centrioles. These data not only help to advance understanding of the human centriole ultrastructure but also advance the understanding of centriolar molecular composition by fitting into the 3D volume centriole/BBs proteins that have been previously structurally resolved (Nazarov et al., 2020). In this way, it is now possible to propose specific functions of each structural element of the BBs/centriole. These functions might be tested by localizing proteins by U-ExM and analyzing carefully their RNAi-depletion phenotypes at the cell level as well as at the electron microscopy level.

### Basal Body Anchoring Process and Orientation

The BB anchoring process leading to transition zone assembly is a crucial step in ciliogenesis, since mutations in genes involved in this process are associated with severe ciliopathies such as Meckel (MKS), Joubert (JBTS), Jeune Nephronophtisis (NPHP), Oro-Facial-Digital (OFD) syndromes. The BB anchoring involves multiple cell components: the distal end of the BB, the plasma membrane and cytoskeletal elements that guide the BB and coordinate the operations**.**

In both *Paramecium* and *Tetrahymena*, BBs are arranged in longitudinal rows, and their polarity is marked by the asymmetrical organization of their associated appendages (Figures 2E,F, Figures 5A,B). Unlike metazoa, there is no centrosome stage in these species, since BBs always remain anchored at the cell surface. New BBs develop from the docked ones; once duplicated, they just have to tilt-up to anchor directly at the cell surface. In mammals, many proteins involved in BB anchoring are located in both centrioles and centriolar satellites, non-membranous, electron-dense spherical cytoplasmic granules. This dual location introduces the possibility that these proteins could have multiple different functions depending on their localization. Importantly, there are no centriolar satellites in ciliates, such as paramecia, making the phenotype observed after RNAi depletion specifically due to the BB pool.

**FIGURE 7:**
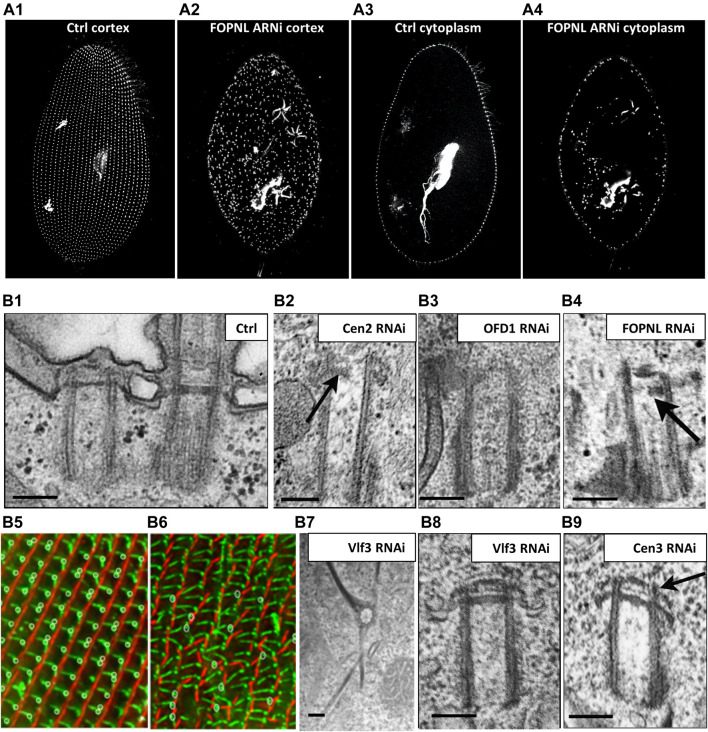
*Paramecium*, a model to study basal body anchoring defects. **(A)** A1-A4: Control *Paramecium* (A1, A3) and FOPNL-depleted *Paramecium* (A2, A4) decorated for basal bodies (ID5 antibodies) observed by confocal at the cell cortex (A1, A3) or at intracytoplasmic level (A2, A4). Note that FOPNL depletion leads to a disorganized pattern of basal bodies at the cell cortex. Unanchored basal bodies are found in the cytoplasm (A4) (reproduced/adapted with permission "Aubusson et al, 2012. Journal of cell science, 125, 4395-440"). **(B)** Transmission electron microscopic images of a basal body doublet anchored at the cell surface. Note that the TZ vary in height between the ciliated basal body and the unciliated one (B1). B2-B4: Depletion of Centrin2 (B2), OFD1 (B3) (reprint from Bengueddach et al, 2017, Cilia 6, 6. doi:10.1186/s13630-017-0050-z), and FOPNL (B4) lead to unanchored basal bodies with defective organization of its distal end (reproduced/adapted with permission Aubusson et al, 2012. Journal of cell science, 125, 4395-4404). B5-B6: immunofluorescence of a control *Paramecium* (B5), or cell depleted for SF-assemblin depleted group-I proteins (B6) from Nabi et al., 2019 with permission, stained for the kinetodesmal fiber (red) and microtubule rootlets (green). Note the disorganization of the basal bodies and mis-orientation of their rootlets at the cell surface (Nabi et al., 2019). Depletion of Vfl3 protein (B7) induces the formation of more than one kinetodesmal fiber indicating rotational polarity defect (reprint from Bengueddach et al, 2017, Cilia 6, 6. doi:10.1186/s13630-017-0050-z). By contrast, the distal end of the basal body is well organized as after depletion of Centrin3 (B9) (reproduced/adapted with permission Aubusson et al, 2012. Journal of cell science, 125, 4395-4404). Scale bars: 250 nm.

Thanks to the precise organization pattern of BBs over the cell cortex, defects in their anchoring are easily recognized not only by the presence of some internal BBs, but also by anomalies in the surface pattern by Immunofluorescence (IF) experiments (Figure 2A, Figure 5A). Using IF in *Paramecium*, we have been able to show that depletions of the conserved distal-end BB proteins (Centrin2, OFD1, FOPNL/FOR20 or CEP90) lead to mispositioned and unanchored BBs (Aubusson-Fleury et al., 2012; Bengueddach et al., 2017; Borgne et al., 2021; Ruiz et al., 2005 Figure 7A1-A4). The close observation by EM of these unanchored BBs revealed that their distal ends, which mimic the unciliated TZ, are incomplete. Whereas depletion of Centrin2 leads to long BBs with an almost complete absence of their distal ends (Figure 7B2), the depletion of either OFD1 or FOPNL shows a partially organized TZ (Figures 7B3,B4), compared to Control depleted cells (Figure 7B1) suggesting that Centrin 2 is acting at the distal end, earlier than OFD1, FOPNL and CEP90 (Ruiz et al., 2005; Aubusson-Fleury et al., 2012; Bengueddach et al., 2017; Borgne et al., 2021). Indeed, this has been demonstrated using paramecia expressing Centrin2-GFP and RNAi-depleted for OFD1, FOPNL or CEP90 and vice-versa. These studies demonstrate that proteins that localize at the distal end of BBs early after BB duplication are critical for both the assembly of the distal end and also for the BB docking event. Interestingly, in mammals, these proteins have also been shown to prevent BB docking by preventing distal appendage formation (Borgne et al., 2021; Kumar et al., 2021) leading to impaired ciliogenesis and, therefore, to ciliopathies (Singla et al., 2010; Chevrier et al., 2016; Bruel et al., 2017). These proteins localize in a 9 fold symmetry at the distal end of the BB at a position where the distal appendages are observed by expansion microscopy (Borgne et al., 2021). To support this assumption, Kumar et al. (Kumar et al., 2021) showed an interaction between CEP90 and CEP83, the proximal distal appendage protein. As in *Paramecium*, OFD1 and CEP90 are recruited on mammalian newborn procentrioles. Altogether these data led us to propose that the complex composed of FOPNL, OFD1 and CEP90 determine the future location of distal appendages (Borgne et al., 2021).

Cytoskeleton elements are essential for guiding and positioning BBs. In *Paramecium*, as previously mentioned, three rootlets are associated with the BB. In *Tetrahymena,* post-translational tubulin modifications of both the transverse microtubule ribbon and the post-ciliary rootlet are required for BB attachment to the cell cortex. For instance, defective tubulin glycylation shortens BB-appendage microtubules and disrupt BB positioning and cortical attachment (Junker et al., 2019). Additional links anchor the two BBs to the striated rootlet (Iftode & Fleury-Aubusson, 2003). In 2013, Jerka-Dziadosz (Jerka-Dziadosz et al., 2013) highlighted by EM the presence of a transient filament, that they called Anterior Left Filament (ALF). This filament is localized at the anterior left of the mother BB; it forms before duplication and disappears as the new BB is anchored at the surface. This filament is also observed in *Tetrahymena* and accompanies BB duplication. Several observations suggest that Centrin3 is required for ALF formation: 1) the presence of ALF is observed in wild type cells but not in Centrin 3 depleted cells; consequently, the new BBs remain attached to their mother BBs and do not dock at the cell surface despite their fully formed distal end (Figure 9B9); 2) Centrin3-GFP localized to the proximal part of the BB, anterior and near the base of the appendages (striated rootlet, transverse microtubule ribbon); and 3) At the EM level, Centrin3 localizes at the base of the ALF (Jerka-Dziadosz et al., 2013). Altogether, these results suggest that the ALF is required for the movement of the duplicated BB toward its docking site.

**FIGURE 8:**
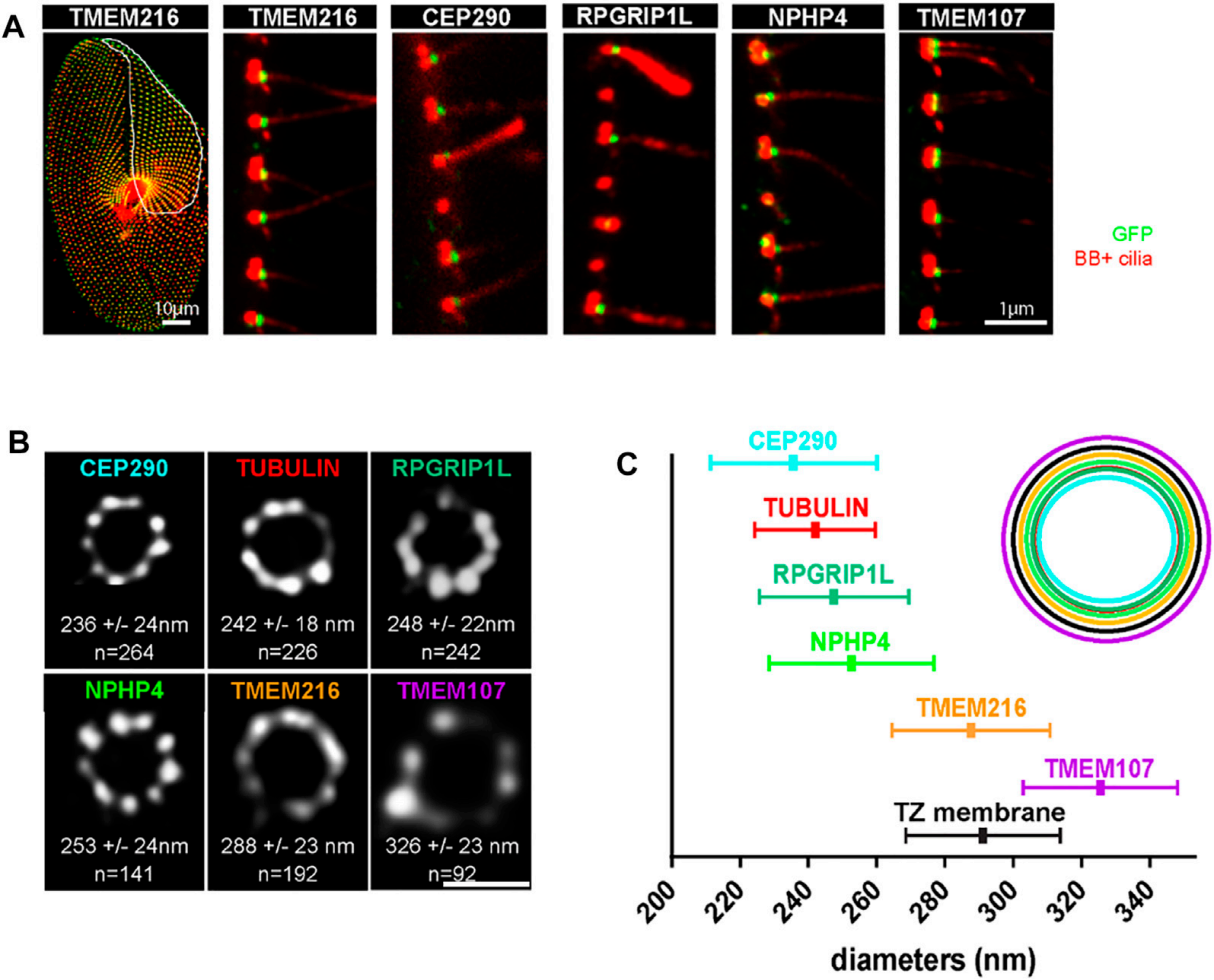
**(A)** Paramecia expressing different TZ proteins fused with GFP. Cells were permeabilized before proceeding to immunostaining by ID5 (decorating BB and cilia in magenta) and a polyclonal anti-GFP (in green). Left panel: surface view of a *Paramecium* expressing TMEM216-GFP. Ciliated basal bodies of the invariant field (encircled in white) are stained by both GFP antibodies and ID5. In the other part of the cells, some basal bodies are labelled only by ID5. Right panels: confocal Z projections of ciliary rows, at the cell margin from TZ protein transformants. TMEM107-GFP, TMEM216-GFP, CEP290-GFP, and RPGRIP1L-GFP are localized only on the distal part of ciliated BBs. In addition, NPHP4-GFP can be observed at the BB proximal part. Note that ID5 antibodies better recognize short cilia. Bars = 10 and 1 μm. **(B)** Representative STED images revealing distinct localization patterns of several GFP-tagged TZ proteins. Cells were labelled with anti-GFP or ID5 (tubulin). A single ring differing in diameter is observed according to the observed protein. The mean diameters (distance between intensity maxima) and the number of BBs analyzed are given beneath each image, 2 replicates. **(C)** Graph (left) showing the mean diameter ± Standard Deviation of each toroid labeled by each GFP tagged TZ protein (see **(B)**. Top right, schema representing the relative position of all toroids with respect to the position of tubulin and the ciliary membrane. Each TZ protein is shown in a different color reproduced from Gogendeau et al, 2020, PLoS Biol 18(3): e3000640. 10.1371/journal.pbio.3000640.

Analyzing *Paramecium* BB cortical organization in the early 1960s, Sonneborn and Beisson demonstrated that the newly formed cortical structures are constrained by the cortical environment existing at the time of their development (Beisson & Sonneborn, 1965; Sonneborn, 1964). They proposed that striated fibers (also called striated rootlet, SR) guide the newly formed BBs on the cortical structure by linking neighboring BBs to each other and to the cell cortex (Galati et al., 2014; Soh et al., 2020). Effectively, depletion of the SR protein DisAp in *Tetrahymena* prevents SR elongation and consequently BB organization at the cell surface (Galati et al., 2014). Similarly, *Paramecium* SRs are shorter and misshapen and BBs are no longer positioned properly at the cell surface after depletion of sub sets of the SR proteins from the SF-Assemblin family ((Nabi et al., 2019) Figure 7B6). Finally, the connection between the SR and the BB allows metachronal ciliary beating for cellular motility (Soh et al., 2020). Mispositioned BBs lacking their associated fibers are observed in *Chlamydomonas* mutated in the Vfl3 gene. This led Koll and collaborators (Bengueddach et al., 2017) to study the localization of Vfl3 protein in *Paramecium*. Interestingly, Vfl3 protein localized transiently during the early steps of BB duplication between the microtubule wall and the proximal end of the SR. This might suggest that Vfl3 is part of the cytoskeleton fibers located between the ALF and the SR (Bengueddach et al., 2017, see Figures 2E, F). Due to the importance of Centrin 3 in the ALF formation and BB positioning and anchoring at the cell surface, we suspect a role of Vfl3 in the BB positioning and anchoring process. As expected, depletion of Vfl3A protein led to mispositioned BBs at the cell surface with some of them being located deep in the cytoplasm. In EM images of unanchored BBS, the distal part appears similar to the distal end of BB observed in Centrin3-depleted cells, suggesting that the unanchored phenotype is not the consequence of a defect of BB distal end formation. Intriguingly, the mispositioned BBs display abnormalities of the associated rootlets (SR, and transverse, and post-ciliary microtubule ribbons) with some BBs showing 2 or more SRs (Figure 7B7, Figure 8B8), while others have none, suggesting that the rotational polarity of the BB is affected in Vlf3 depleted cells. Similar results concerning the role of Vfl3 in the assembly of BB appendages in planarians has also recently been described (Basquin et al., 2019). All these results suggest that the function of this protein in defining the proper microtubular triplet for assembly of appendages is conserved throughout evolution.

### Structure/Function of the Transition Zone (TZ)

In *Paramecium*, docked non-ciliated and docked ciliated BBs are present in the cortex of the same cell. Cilia form on docked BBs throughout the cell cycle (Gogendeau et al., 2020), and it is this BB docking event that leads to the formation of the TZ of the new cilium.

Defective TZ formation has been observed in *Tetrahymena* after co-depletion of POC5 and the POC5-like Sfr1 protein (Heydeck et al., 2020) despite what appears to be correctly anchored BBs. In these co-depleted cells, cilia formation is impaired, suggesting that there is a requirement for Poc5 in building the distal end of BBs. In addition to this defective TZ phenotype, depletion of POC5, Sfr1 or their co-depletion leads to too many rows of BBs (Heydeck et al., 2020). How POC5 may affect these two processes is not yet clear and further studies are required to shed light on those phenotypes.

The TZ acts as a gate-keeper for proteins entering and leaving the cilium, thereby controlling a specific ciliary composition (Garcia-Gonzalo and Reiter, 2017). In *Paramecium* the TZ is organized in three distinct plates defined as terminal, intermediate and axosomal plates (Dute & Kung, 1978; Tassin et al., 2015) see Figure 1F. As in other species, transition fibers and Y-links are present in *Paramecium*. With the exception of CEP164, the characterized mammalian transition fiber components are poorly conserved in *Paramecium*. However, members of the different modules known in various model organisms to cooperate in the assembly of the TZ are conserved in *Paramecium* where they localize only at ciliated BBs (Figure 8A) (Gogendeau et al., 2020).

STED (stimulated-emission-depletion) microscopy of paramecia expressing GFP-tagged TZ proteins demonstrates that these conserved TZ proteins are organized, as in other cell models, in a nine fold symmetry (Figures 8B,C). They are recruited to the TZ as soon as the cilium starts to grow. This molecular recruitment is accompanied by an elongation of the TZ, which is considered as a structural maturation (Gogendeau et al., 2020). We propose that Intraflagellar Transport (IFT) is required for both the construction of the cilia but also for the structural elongation of the TZ.

Deciliation is a process conserved from unicellular animals to mammals that leads to cilia/flagella shedding. Deciliation has been largely described by electron microscopy in the fallopian tube in mammals and birds during the menstrual cycle according to the hormonal cycle as well as in the upper airway following smoke inhalation. This process is not restricted in mammalian multiciliated cells, since ciliary shedding seems the predominant mode of ciliary loss during IMCD3 (mouse Inner Medullary Collecting Duct-3) cell cycle (Mirvis et al., 2019). Unicellular organisms such as *Chlamydomonas*, that severs its flagella upon stress induction (for review see (Quarmby, 2004; Quarmby, 2009)), provides a model to study the mechanism of deciliation. The induction of stress leads to an influx of calcium at the level of the TZ that will induce the release of calcium from internal stores. The sequestration of calcium by a calcium binding protein appears to activate the severing machinery.

The ciliary shedding always occurs at the boundary between TZ and the axoneme. This observation led Gogendeau and collaborators (Gogendeau et al., 2020) to study the involvement of MKS-NPHP module proteins in the control of ciliary shedding at the TZ in *Paramecium*.

The functional analysis of these five TZ-proteins (TMEM-216, TMEM107, CEP290, RPGRIP1L, NPHP4) show that they play significant and antagonist functions in cilia shedding. Depletion of either TMEM107 or TMEM216 protein lead to a constant ciliary shedding, which occurs at the level of the axosomal plate, and broken cilia were recovered in the paramecia culture medium. This ciliary shedding is followed by ciliary regrowth, consistent with the results of the transcriptomic analysis of TMEM216 depleted cells that show an upregulation of ciliary genes with half of them being differentially regulated during the reciliation process. Increase of the ciliary beating forces exacerbate the deciliation process, which is prevented by RNAi of DNAH2 (an inner arm dynein required for ciliary beating). Gogendeau and collaborators (Gogendeau et al., 2020) propose that the ciliary beating movement breaks the cilia at the level of the axosomal plates when weakened by TMEM107 or TMEM216 depletions. In contrast, depletion of NPHP4, CEP290 or RPGRIP1L make cilia resistant to Ca^2+^/EtOH-induced deciliation (Gogendeau et al., 2020), unless permeabilized by Triton X-100 and treated with dibucaine or Ca^2+^. Altogether this study suggests that an influx of Ca^2+^ required for deciliation is missing in the cells depleted of NPHP4, CEP290 or RPGRIP1L.

Altogether, this study provides evidence of the conserved function of TZ proteins in ciliary gating and the first evidence for a role of conserved TZ proteins in the deciliation process. In addition, this study opens new directions for understanding motile cilia physiology in mammalian cells.

### *Paramecium,* a Cell Model to Study Primary Ciliary Dyskinesia Candidate Genes

Primary ciliary dyskinesia (PCD) is a rare genetic disease affecting between 1 in 10,000 or 1 in 20,000 individuals worldwide. It is characterized by recurrent respiratory tract infections, caused by motility defects in cilia and flagella. Ineffective cilia movement results in a limited mucociliary clearance in the upper and lower respiratory tract leading to various chronic infections and infertility. PCD is also associated with situs inversus in about 50% of cases leading to the Kartagener syndrome (Ortega et al., 2007).

More than 40 mutated genes have been found in PCD, affecting structural components of the axoneme such as dynein arms, cilia radial spokes, N-DRC (nexin–dynein regulatory complex), and also cytosolic factors required for dynein arm assembly and transport. Despite the progress in expanding the identification of mutations for motile cilia ciliopathies (Horani et al., 2014), in about ¼ of patients the causal gene is not yet known. It is known that some mutated genes, such as DNAH11, impaired cilia motility without impairing axonemal structure, which make the mechanism of impairment complex to understand. In addition, some mutations are only found in one or two families, providing sparse data for the identification of the causal mutations among the polymorphism. For these reasons, the development of model organism allowing a rapid functional analysis of the candidate genes is important.

*Paramecium* with its thousands of motile cilia at its surface is a powerful model to study genes mutated in PCD. Phenotypes are observable as soon as the first cell division after mutagenesis. The swimming behavior and ciliary beat frequency can be recorded, and defects in cilium organization can be visualized by immunofluorescence staining or electron microscopy/tomography. Furthermore, complementation of the RNAi gene knockdown by ectopic expression of the orthologous human gene can be performed using a synthetic gene optimized for *Paramecium* codon usage. Therefore, through functional complementation, the effect of human mutations accounting for PCD in patients can be directly assessed upon expression in *Paramecium*. Alternatively, the human mutation could be also introduced into the *Paramecium* orthologous gene to study the mutation phenotype. Preliminary results where patient mutations were mimicked in paramecia (data not published) build confidence for the use of this model to validate the effect of mutation on pathology when genomic approaches reach their limits to discriminate pathologic mutations to from neutral polymorphism. Using these approaches we had been able to demonstrate in *Paramecium* the involvement of three candidate genes in ciliary motility (Fassad et al., 2018; Thomas et al., 2020).

In conclusion, several features of *Paramecium* contribute to its appeal as a model for ciliary beating, sensory function and ciliogenesis. The large number of cilia present in *Paramecium* makes biochemical and powerful proteomic analyses possible, even when searching for low abundance proteins. When we also apply RNAi silencing, functional complementation, GFP-tagging and subcellular protein localization, we can glean a great deal of information about the trafficking of ciliary proteins and the structure and function of motile cilia. Moreover, the large number of basal bodies with these cilia together with the high conservation of the ciliary structure throughout evolution help us to decipher the structure of BBs, the TZ and cilia using high resolution electron microscopy (cryo-tomography, FIB-SEM and Cryo-FIB) that can inform other cilia systems.

Other physical features of *Paramecium* that should not be overlooked are the highly organized longitudinal rows and orientation of BBs that make it relatively easy using immunofluorescence or low resolution electron microscopy to spot deviations due to molecular manipulation or mutation. Likewise, swimming patterns broadcast whether ciliary positioning and function are normal, facilitating studies of the effects of various manipulations of ciliary function. Therefore, *Paramecium* serves as a powerful and efficient model to analyze not only mutations affecting cilia motility (PCD) but also non motile ciliary defects.

Ciliary motility depends upon ion channels, many of which are specific to the cilia. Several of them are in low abundance. Therefore, the ability to identify the proteins of the cilia using proteomics becomes more powerful when their function can also be studied using electrophysiology of ciliated and deciliated cells. Adding to the value and context of recordings from *Paramecium* is the long history of electrophysiological studies, which help with our explorations of the mechanisms underlying control of ciliary beating and sensory functions.

Finally, in mammals functional divergence of core BB/ciliary proteins have been described (Yadav et al., 2016; Siller et al., 2017; Wiegering et al., 2018) in different ciliated tissues. Analysis of the function of these conserved proteins in evolutionary distant organisms such as *Paramecium* uncover conserved and divergent features in cilia assembly, which is a pre-requisite for understanding tissue specific variations observed in mammalian tissues.
